# Identification and analysis of conserved pockets on protein surfaces

**DOI:** 10.1186/1471-2105-14-S7-S9

**Published:** 2013-04-22

**Authors:** Marco Cammisa, Antonella Correra, Giuseppina Andreotti, Maria Vittoria Cubellis

**Affiliations:** 1 Department of Biology, University "Federico II", Via Cinthia, 80126, Naples, Italy; 2 Istituto di Chimica Biomolecolare -CNR, Comprensorio Olivetti, 80078, Pozzuoli, Italy; 3 Istituto di Biostrutture e Bioimmagini-CNR, Via Mezzocannone, 80134, Napoli, Italy

## Abstract

**Background:**

The interaction between proteins and ligands occurs at pockets that are often lined by conserved amino acids. These pockets can represent the targets for low molecular weight drugs. In order to make the research for new medicines as productive as possible, it is necessary to exploit "in silico" techniques, high throughput and fragment-based screenings that require the identification of druggable pockets on the surface of proteins, which may or may not correspond to active sites.

**Results:**

We developed a tool to evaluate the conservation of each pocket detected on the protein surface by CastP. This tool was named DrosteP because it recursively searches for optimal input sequences to be used to calculate conservation. DrosteP uses a descriptor of statistical significance, Poisson p-value, as a target to optimize the choice of input sequences. To benchmark DrosteP we used monomeric or homodimer human proteins with known 3D-structure whose active site had been annotated in UniProt. DrosteP is able to detect the active site with high accuracy because in 81% of the cases it coincides with the most conserved pocket. Comparing DrosteP with analogous programs is difficult because the outputs are different. Nonetheless we could assess the efficacy of the recursive algorithm in the identification of active site pockets by calculating conservation with the same input sequences used by other programs.

We analyzed the amino-acid composition of conserved pockets identified by DrosteP and we found that it differs significantly from the amino-acid composition of non conserved pockets.

**Conclusions:**

Several methods for predicting ligand binding sites on protein surfaces, that combine 3D-structure and evolutionary sequence conservation, have been proposed. Any method relying on conservation mainly depends on the choice of the input sequences. DrosteP chooses how deeply distant homologs must be collected to evaluate conservation and thus optimizes the identification of active site pockets. Moreover it recognizes conserved pockets other than those coinciding with the sites annotated in UniProt that might represent useful druggable sites. The distinctive amino-acid composition of conserved pockets provides useful hints on the fundamental principles underlying protein-ligand interaction.

**Availability:**

http://www.icb.cnr.it/project/drosteppy/

## Background

Proteins are large molecules characterized by complex structures whose main function is to keep relatively small active sites in good shape. Indeed a precise 3-D architecture is necessary to grip ligands efficiently. A protein surface represents an irregular landscape, rich in pockets and clefts. There can be various binding sites in a single protein and ligands can be as small as ions or large polymers and can function as substrates, inhibitors or allosteric modulators.

Several methods predict ligand binding sites or functional important residues on protein surfaces. Some, such as CastP [1,2] or SURFNET [3], exploit geometric properties. Others, such as PDBinder [4], are knowledge-based. Others again, such as ConSurf [5], ConCavity [6], LIGSITE [7], Crescendo [8], combine 3D-structure and evolutionary sequence conservation.

To explore the pockets on protein surfaces we developed DrosteP, a program which assesses the conservation of the residues lining the pockets on a protein surface. DrosteP requires that the pockets on the surface of a protein with known 3D-structure are identified with the program CastP [1,2] and that conserved amino acids are identified aligning multiple homologous sequences.

Any method relying on conservation mainly depends on the choice of the input sequences and the originality of our approach derives from the fact that we specifically address this point. DrosteP uses a descriptor of statistical significance, Poisson p-value, as a target to optimize the choice of input sequences. We demonstrated that deciding how far one should go to collect homologs to calculate residue conservation influences the precision of active site pocket identification.

Given a protein structure, the output of CastP analysis and a set of homolgous sequences, DrosteP supplies the most conserved pocket as well as with the identity of the other conserved pockets. For each pocket the amino acid composition is provided in order to facilitate the identification of the active site and of other druggable pockets

## Methods

### DrosteP algorithm

Given the sequence of a protein with known 3D-structure, we gather N homologous sequences and we put them in order by increasing e-value. We build different alignments in FASTA format including sequences with higher e-value recursively. For each alignment j (1 < j < N-1) we identify conserved amino acids and we calculate the total number of atoms belonging to conserved amino acids, TCAj. Under standard conditions the program uses 50 sequences form Uniprot/Swiss-Prot, but any alignment in FASTA format of length N can be chosen. In order to avoid any arbitrary choice of a scoring system, DrosteP identifies amino acids that are 100% identical, but the percentage is lowered if no amino acid completely conserved are found in the multiple alignment. We ran CastP [1,2] on the protein structure and get the atoms and amino acids lining each pocket.

We calculate the observed pocket conservation OPCij i.e. the observed number of atoms lining the pocket i and belonging to amino acids conserved in alignment j. We estimate the expected pocket conservation EPCij i.e. total number of atoms belonging to conserved amino acids in the alignment j (TCAj) multiplied by the number of atoms lining pocket i (PAi) divided by the total number of atoms of the protein(TA) (EPCij = TCAj × PAi/TA).

We choose the alignment j which provides the OPCij with the lowest p-value (Poisson probability p = e^-EPCij ^EPCij^OPCij^/OPCij !).

Once the best alignment is chosen, we identify the pockets enriched in conserved amino acids (OPCij/EPCij > 1) at a level of statistical significance, Poisson p-value, lower than 0.05. From now on these will be defined as conserved pockets. The pocket enriched in conserved amino acids and associated with lowest p-value is predicted to be the active site pocket.

To validate the method for identification of active sites based on pocket conservation, we used two groups of proteins with known 3D-structure whose active site had been annotated in Uniprot/Swiss-Prot.

A first set comprises human monomeric proteins. A second set comprises human homodimers, but we restricted the analysis to cases where two chains are found in the crystallographic asymmetric unit. Using these criteria we collected 460 monomers and 165 homodimers.

We downloaded all the unique sequences associated to each PDB structure from Consurf-DB [9]http://consurf.tau.ac.il/.

To test the effect of redundancy, we run skipredundant [10] to obtain the cluster of sequences that have less than 70% identity pair wise.

### Analysis of pockets

Pocket size was measured running CastP. To calculate amino acid composition, we summed all the atoms of a given amino acid lining the pockets and divided the result by the total number of the atoms in the pockets. This calculation was carried out considering all pockets or considering separately conserved (OPCij/EPCij > 1 and p < 0.05) or non conserved pockets (OPCij/EPCij < 1 or p > 0.05).

Prosite patterns were downloaded from ftp://ftp.expasy.org/databases/prosite/. Patterns containing only conserved Cys were excluded, for the remaining patterns, only amino acids completely conserved were considered to calculate amino acid composition.

### Statistical analysis

One-Way Analysis of Variance for Independent Samples was performed to evaluate statistical significance of differences observed when comparing DrosteP and Consurf and when analyzing amino acid or atom type abundance in conserved pockets.

## Results

### Identification of conserved pockets and active sites

Most programs identify functionally important residues in protein structures. We addressed a slightly different problem, that is, we tried to identify the active site pocket in a protein.

It can be expected that active site pockets contain some functionally active residues, but they also contain residues that are not directly involved in ligand binding. For some studies it is preferable to identify active site pockets. For instance it was demonstrated that mutations occurring in the active site pocket, but not affecting residues directly binding the ligand (as seen in the x-ray structure of protein-ligand complex) can be pathological and prevent the use active site directed drugs [11]. Moreover the definition of an active site pocket is required to bind small ligands to proteins by in silico docking.

Our method, DrosteP, complements a program which finds pockets on protein surfaces. For the preliminary task of pocket identification we chose CastP [1,2], but in principle other programs might be employed. Once pockets on the surface of a protein with known 3D-structure have been identified, homologous sequences are collected and aligned in FASTA format. Combining results obtained by CastP and the alignment, DrosteP assesses the conservation of the residues lining the pockets.

The number of conserved residues depends on the depth reached in gathering homologs. For this reason DrosteP starts with the first two sequences of the alignment and recursively adds aligned sequences.

For each number j of homologous sequences included in the alignment, DrosteP calculates observed (OPCij) and expected (EPCij) conservation for each pocket i detected by CastP and chooses the alignment j which provides the lowest Poisson p-value. Once chosen the optimal alignment, DrosteP allows to distinguish between non conserved and conserved pockets and to select the most conserved pocket in the latter group, which is predicted to be the active site pocket.

DrosteP precision was calculated counting the numbers of the most conserved pockets that include (TP) or do not include (FP) the active site residues annotated in Uniprot/Swiss-Prot. The tests were carried out running CastP on monomeric or homodimeric human proteins and aligning the sequence of each one to homologous proteins from other species.

**Figure 1:**
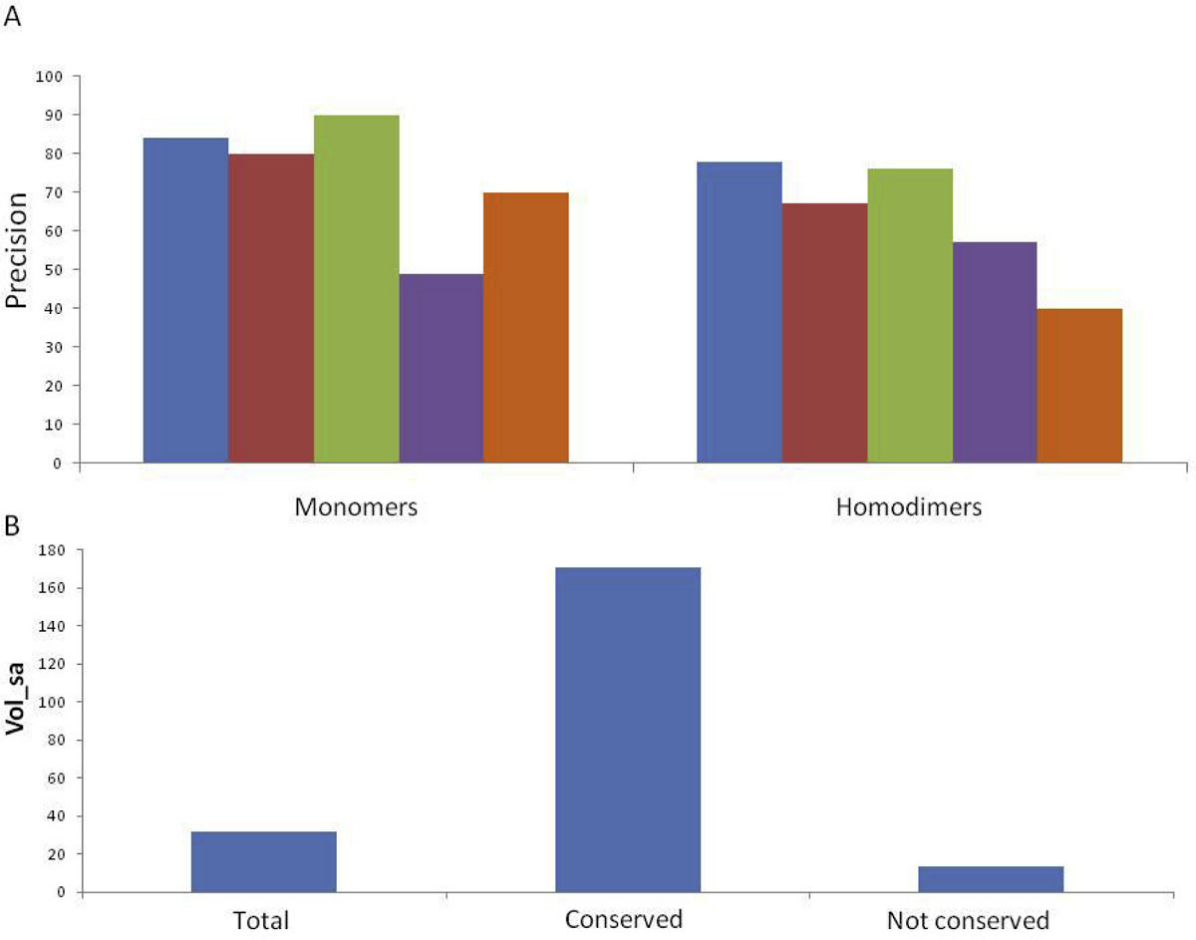
**Prediction of active sites**. Panel A: The percentage of correctly predicted active sites in human monomeric and homodimeric proteins: blue bars refer to predictions relying on the identification of the most conserved pocket (DrosteP protocol), red bars refer to predictions relying on the identification of the largest pocket, green bars refer to predictions relying on the identification of the largest among the conserved pockets, violet bars refer to predictions relying on the identification of the most conserved pocket without DrosteP optimization, orange bars refer to predictions based on Jensen Shannon Divergence score. Panel B: Solvent accessible volume in protein pockets: total, conserved or non conserved ones.

The precision of our method (blue bars in Figure 1A) is directly comparable to CastP (red bars in Figure 1A), which finds pockets, measures their volumes and identifies the active pocket as the largest pocket [12,13].

We obtained higher precision on homodimers with the method based on sequence conservation (78% versus 67%) and comparable precisions on monomers (84% versus 80%). The size of conserved pockets is variable but, in general they are larger than the non conserved pockets (Figure 1B). We can detect active sites of monomeric proteins with higher precision if we consider both conservation and size. In fact the active site of monomeric proteins coincides with the largest among the conserved pockets in 90% of the monomeric cases. On the other hand in the case of homodimers, precision drops to 76% when the largest pocket among the conserved ones is considered (green bars in Figure 1A).

Recent methods that predict active site pockets or functional residues exploiting evolutionary conservation, ConSurf [5] and LIGSITE [7], rely on Consurf-DB [9], a database where sequence homologs of each of the PDB entries were collected and aligned using standard methods.

The novelty of our method consists in the optimization of the list of homologous sequences to be used to calculate conservation. To test this point specifically, we detected the most conserved pocket either considering the complete list of homologous sequences stored in Consurf-DB (violet bars in Figure 1A) or the optimal list obtained by truncation with DrosteP (red bars in Figure 1A). We found that DrosteP raises precision from 49% to 84%, for monomer and from 57% to 78% for dimers. For a comparison, we also identified the active site pocket as the one which contains the most conserved residue predicted by Jensen Shannon Divergence (JSD) score [14] and the results are shown in Figure 1A by orange bars.

To test the effect of redundant sequences in the alignment, we created for each protein a set from Consurf-DB where no pair of homologous sequences had more than 70% identical residues. The overall precision of DrosteP with and without redundant sequences was 81% and 76% respectively.

Notwithstanding the differences of the outputs, we compared the accuracy of DrosteP to that of another program based on sequence conservation, ConSurf [5,15]. We considered the residues to which ConSurf assigns the highest score as the active site pocket. We counted the number of residues in the active site coinciding with those annotated in Uniprot/Swiss-Prot and considered them as true positive. With this definition DrosteP outreaches ConSurf as far as accuracy is concerned and true negative rate although true positive rate is lower (table 1).

**Table 1:** Comparison between DrosteP and Consurf predictions

	DrosteP	Consurf	p-value
**Accuracy**	96.07 ± 0,47	79.31 ± 0.40	< 0.0001
**Precision**	6.21 ± 1,99	1.95 ± 0.60	< 0.0001
**Recall**	54.15 ± 11,17	90.85 ± 5.06	< 0.0001
**True negative rate**	96.28 ± 0.59	79.26 ± 0.41	< 0.0001
**Negative predictive value**	99.77 ± 0.13	99.94 ± 0.05	0.0130

### Extended active sites

Generally speaking, direct identification of active sites and functional residues relies on experiments of mutagenesis or chemical modification and provides only a limited number of the residues involved in substrate binding. In some fortunate cases a more detailed view of the active site is possible because the protein structure has been solved in the presence of a substrate analogue. But even in these cases the view can be incomplete. In fact natural substrates are often molecules larger than those co-crystallized with the enzymes. The knowledge of the wide-ranging active site, that is the surface which is in touch with the natural substrate or ligand, is useful in many cases. For instance it is needed to evaluate the effect of protein modifications in human diseases because mutations occurring in the active site pocket, even if not directly involved in catalysis, are almost inevitably harmful and unsuitable for therapy with chaperones [11]. Pharmacological chaperones which represent a novel and promising approach for the cure of many diseases, are typically reversible inhibitors used at low concentration to stabilize the pathological forms of a protein [16] and need a functional active site to bind.

DrosteP is very useful to extend the active site and cover all the surface which is in contact with the natural substrate or ligand. We propose to start from the most conserved pocket and enlarge it by addition of the contiguous conserved pockets. At the present this procedure is manual but, in principle, it could be made automatic.

**Figure 2:**
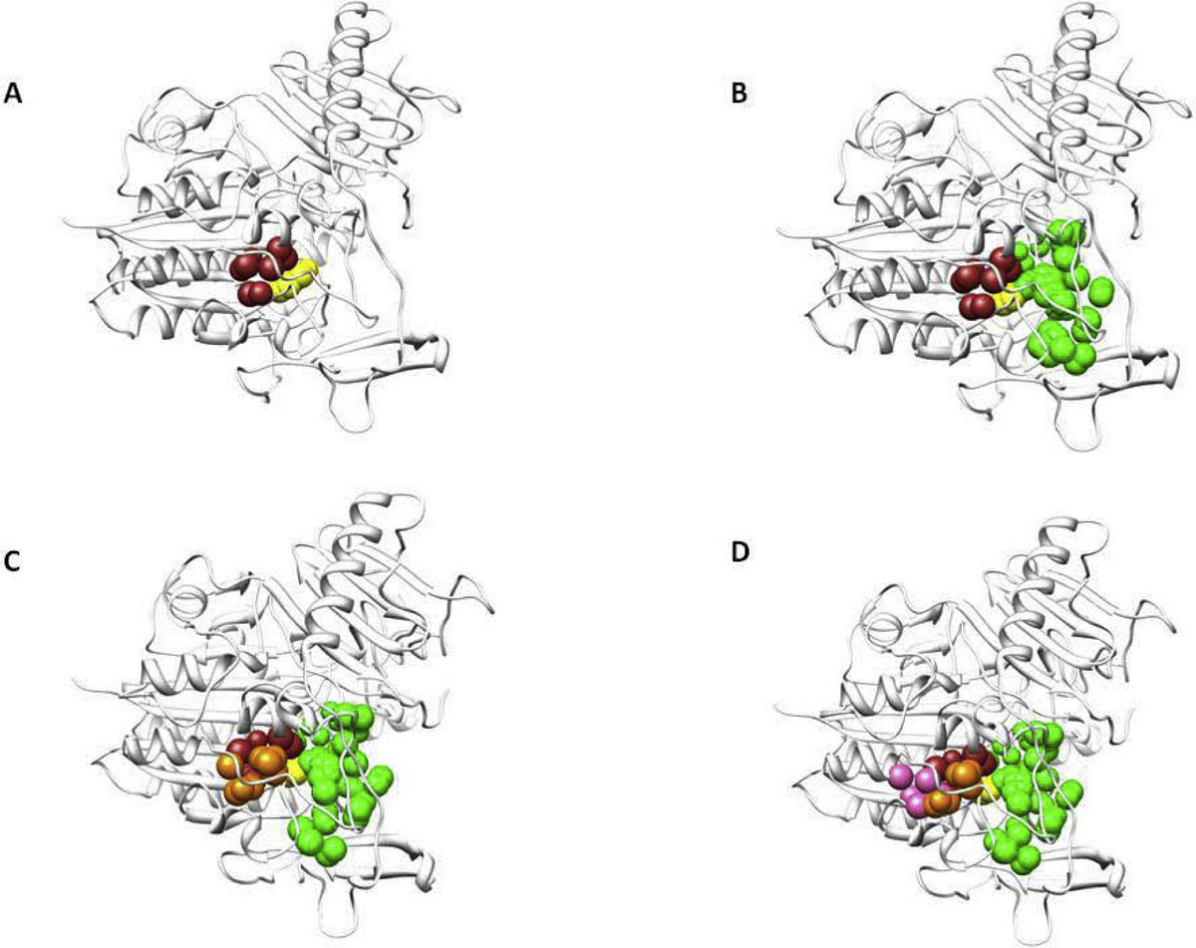
**The composite active site of arylsulfatase B**. The structure of arylsulfatase B is shown as a ribbon. Panel A the active site residue K145 is shown in yellow, the atoms lining the most conserved pockets are shown as brown spheres. Adjoining pockets are added in order of decreasing conservation starting from panel B where the atoms are shown as green spheres, to Panel C, where the atoms are shown as orange spheres and to Panel D, where the atoms are shown as pink spheres.

As an example we will describe the case of arylsulfatase B (Uniprot:ARSB_HUMAN), an enzyme which catalyzes the hydrolysis of the 4-sulfate groups of the N-acetyl-D-galactosamine 4-sulfate units of chondroitin sulfate and dermatan sulfate [17]. Defects in arylsulfatase B are the cause of mucopolysaccharidosis type 6 [MIM:253200] [18]. Our method identifies a relatively small pocket (brown in Figure 2) lined by atoms belonging to residues R95, L99, M142, K145, W146, L148, Y235 as the most conserved one. We learn from the annotation in Uniprot that K145 (yellow in Figure 2) is the binding site for the substrate and that mutations of R95, M142, W146 represent some of the molecular defects which lead to mucopolysaccharidosis type 6. This site can be extended to include another 3 conserved pockets, (in order of decreasing conservation, green, orange and pink in Figure 2) which are contiguous to the most conserved one. The composite active site covers most of the functional or disease-associated residues and is large enough to accommodate the natural substrates.

### Properties of conserved pockets

**Figure 3:**
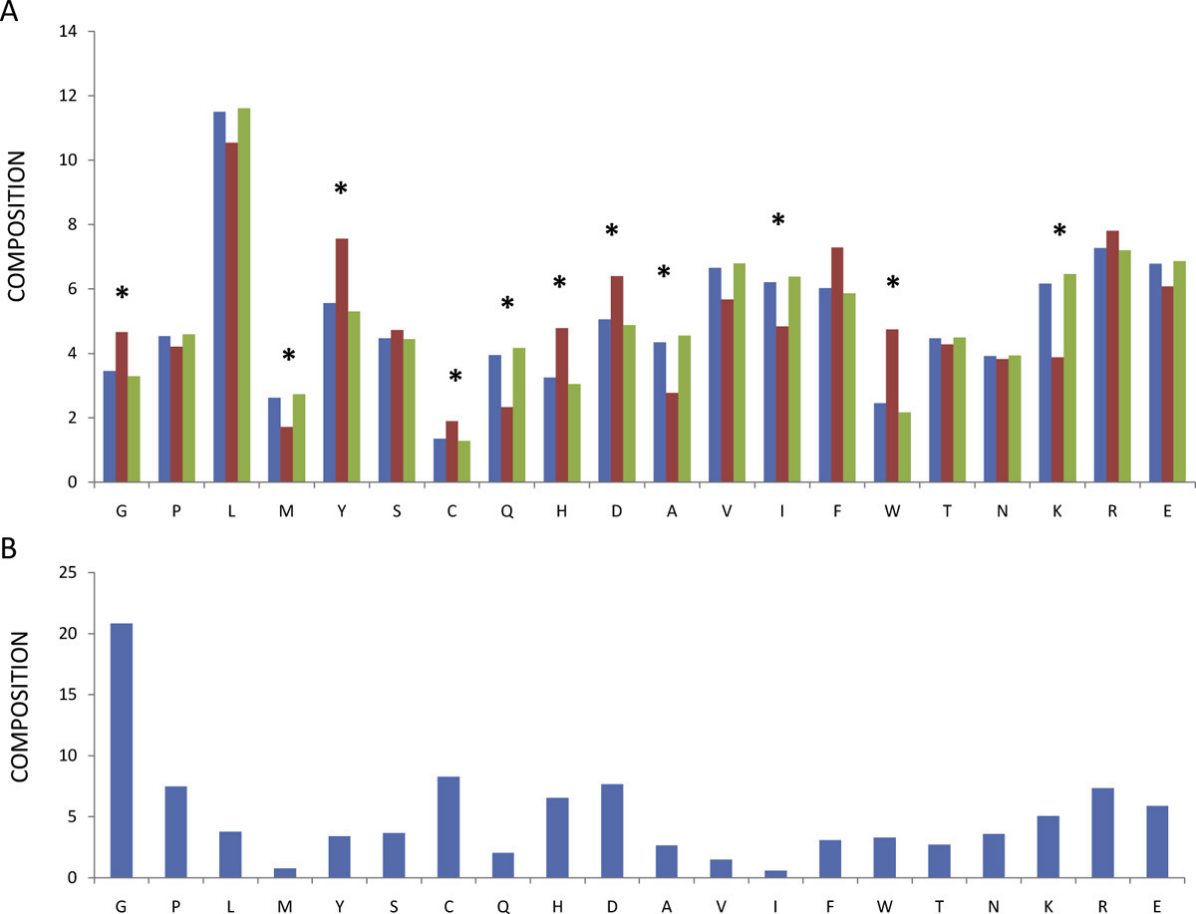
**Amino acid abundance in pockets and in Prosite patterns**. Panel A: Blue bars refer to relative abundance of amino acids in pockets, red bars refer to relative abundance in conserved pockets, green bars refer to relative abundance in non conserved pockets. An asterisk marks those cases where the difference between conserved and non conserved sites is statistically significant (p < 0.05). Panel B: Amino acid relative abundance in Prosite Patterns.

CastP produces a list of the amino acids in a pocket with a detailed description of all the atoms exposed to solvent. This allows us to calculate the amino acid composition. Although the composition of individual pockets is variable, the abundance of some amino acids differs between conserved and non conserved pockets significantly as shown in Figure 3A. This suggests that not only active sites, but also other conserved pockets on the protein surface play important roles.

In order to discuss this effect we added in Figure 3B the amino acid composition calculated on conserved residues found in the Prosite patterns [19]. A pattern describes a short, contiguous stretch of protein which is conserved in a protein family either for functional or for structural reasons.

Gly is a small amino acid whose abundance in proteins from vertebrates is similar to that of Ala and Ser. Nonetheless it is overrepresented with respect to Ala and Ser among the amino acids conserved in Prosite patterns. This finding suggests that peculiarities other than the small size bestow a special role to Gly. Limiting the analysis to the protein surface, we observe that Gly is more abundant in conserved than in non conserved pockets. The absence of a side chain, the possibility to adopt different dihedral angles as well as its requirement in beta turns, might be the cause of its overrepresentation in conserved pockets.

Charged amino acids are relatively abundant among residues conserved in Prosite patterns. It is common knowledge that His plays a pivotal role in many active sites and it is not surprising that it is preferentially seen in conserved pockets. Less expected is the finding that the only other charged amino acid preferentially found in conserved pockets at a statistically significant level is Asp.

**Figure 4:**
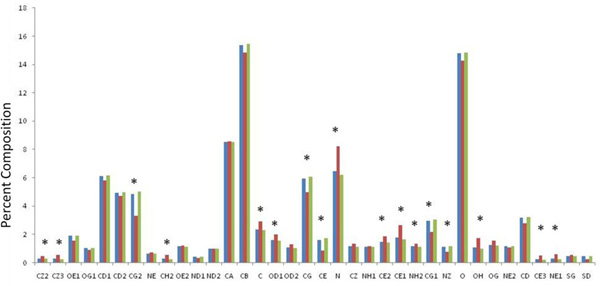
**Atom type abundance in pockets**. Panel A: Blue bars refer to relative abundance of atom types in pockets, red bars refer to relative abundance in conserved pocket, green bars refer to relative abundance in non conserved pockets. An asterisk marks those cases where the difference between conserved and non conserved sites is statistically significant (p < 0.05).

Leu, Phe, Tyr and Trp (Figure 3B) have a similar frequency in Prosite patterns although Leu is by far more abundant in proteins from vertebrates. With exception of Tyr, these residues are found in the core more commonly than on surface of native proteins [20]. On the protein surface they could contribute to the stabilization of protein ligand interaction by means of hydrophobic effect [21]. For this task, in principle, both aromatic and aliphatic amino acids are suited, yet we observe that the former ones are preferentially found in conserved pockets (Figure 3A). Consequently aromatic amino acids, and in particular of Tyr and Trp, should play a special role. Possibly cation-pi bonding, aminoaromatic and stacking interaction play an important role in protein functional sites. In order to clarify specific contributions in ligand binding, we examined the distribution of specific atom types (Figure 4).

Main chain amide nitrogen is by far the atom type with the highest preference for conserved pockets. Generally speaking we observed that hydrogen bond donors are more abundant in conserved pockets than hydrogen bond acceptors. The relative abundance of NE1 from Trp and OH from Tyr which can contribute with hydrogen bonding, suggests that aromatic interaction provides selectivity as well as stability to protein ligand interactions.

Our results rely on inference from *in silico *analysis of structural databases and provide an understanding of the role of specific amino acids and atom types in molecular recognition. Other studies addressed the same problem from different points of view.

Koide and Sidhu summarized their findings with a brilliant title "The Importance of Being Tyrosine: Lessons in Molecular Recognition from Minimalist Synthetic Binding Proteins"[22]. They exploited synthetic antibody libraries and demonstrated that "antigen-binding sites that are rich in Tyr and Ser are highly specific and functional, but the addition of Gly can improve function." They also observed that "surfaces containing Trp limited to CDR-H3 are also fairly specific" [23].

Vajda and coworkers [24] exploited another approach, computational solvent mapping, to identify hot spots for protein ligand interaction [24]. They chose ten important pharmaceutical targets and they found almost invariably aromatic residues, preferentially Tyr, among the residues important for ligand binding.

**Figure 5:**
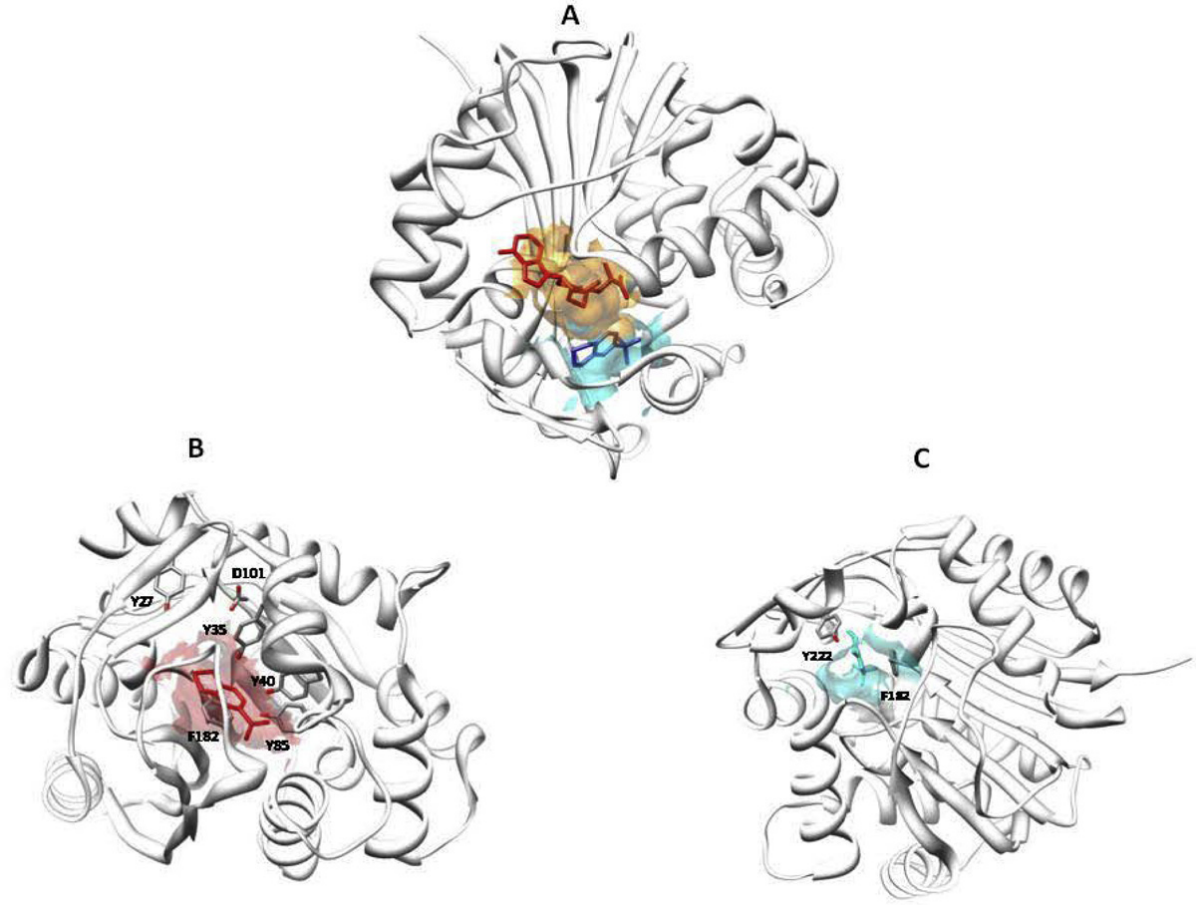
**The active site of Phenylethanolamine N-methyltransferase**. The stucture of a monomer of Phenylethanolamine N-methyltransferase is shown as a ribbon. Panel A: S-adenosyl-L-homocysteine is shown with red sticks in its pocket whose surface is in pale red. 1,2,3,4-tetrahydroisoquinoline-7-sulfonamide is shown in blue in its pocket whose surface is in pale blue. Panel B: The pocket for S-adenosyl-L-homocysteine with Y35, Y40, Y85, Y27 and D101 shown with sticks coloured by atom types. Panel C: The pocket for 1,2,3,4-tetrahydroisoquinoline-7-sulfonamide with F182 and Y222 shown with sticks coloured by atom types.

A good example of the special role played by Tyr and in general by aromatic residues in binding pockets is offered by Phenylethanolamine N-methyltransferase (PNMT), an enzyme implicated in the biosynthesis of adrenaline. The structure of PNMT has been solved in the presence of one its substrates, S-adenosyl-L-homocysteine (AdoHcy) and of a potent inhibitor, 1,2,3,4-tetrahydroisoquinoline-7-sulfonamide by Grunewald and coworkers [25] (pdb:1hnn). The same authors observed that the active site is " surrounded by a constellation of five tyrosines and two phenylalanines" (we quote their exact phrase because we liked the analogy with stars very much). PNMT active site is a crevice which can be subdivided into two adjoined pockets. The pocket for AdoHcy (shown in orange in Figure 5A) is conserved, whereas that for 1,2,3,4-tetrahydroisoquinoline-7-sulfonamide (shown in cyan in Figure 5A) is not. In the pocket for AdoHcy three of the five tyrosines (Tyr35, Tyr40, and Tyr85) form hydrogen bonds with the carboxylate group of the substrate (Figure 5B). The adenosine moiety is blocked between Tyr 27 and Phe 182 and ribose interacts with Gly 81 and forms hydrogen bonds with Asp 101. In the other pocket, the tetrahydroisoquinoline ring is blocked by a face to face interaction with Phe 182 that adopts a positive phi angle [26] and a face to edge amino-aromatic interaction between Tyr 222 and the saturated nitrogen heterocycle (Figure 5C).

Fused ring systems are frequently encountered in molecules in use as approved or experimental pharmaceutical drugs. The layout which requires a face to face interaction with an aromatic amino acid and face to edge p-X interaction, where X is an heteroatom, might provide a preferential drug binding mode.

## Conclusions

Each protein in a cell is committed in multiple interactions and its surface is richly carved to fulfill this requirement. In this contest it is reductive to refer to one specific pocket as the active site. The active site can be a hive shaped concave opening. The primary site harboring catalytic residues can be surrounded by other pockets forming a composite surface which accommodates the substrate. Separate pockets can be exploited to bind allosteric ligands. Active sites and allosteric sites are meant to bind natural ligands. In addition to these, other druggable pockets could exist which are not occupied under physiological conditions and could be exploited to bind drugs. In order to find hints on the fundamental principles underlying protein-ligand interaction we need to analyze large number of cases and derive statistically significant conclusions. Identification of active sites is difficult and requires the intervention of an expert human eye. On the other hand it is feasible to define conserved pockets precisely and, utilizing such a definition, it is possible to search a database of proteins with known 3D-structure and gather data.

We studied 3,635 non conserved and 460 conserved pockets and a total of 57,815 atoms. Since most conserved pockets are exploited to bind natural ligands, the residues or the atom types preferentially exposed in conserved pockets should be the most suitable for molecular recognition. Three amino acids Tyr, Trp and Gly as well as the main chain amide nitrogen are particularly abundant in conserved sites. This finding is useful when looking for druggable sites on a protein and when scoring poses obtained with in silico ligand docking.

## Competing interests

The authors declare that they have no competing interests.

## Declarations

The publication costs for this article were funded by Telethon - Italy (Grant no. GGP12108) to MVC and GA
